# Comprehensive prediction of potential spatiotemporal distribution patterns, priority planting regions, and introduction adaptability of *Elymus sibiricus* in the Chinese region

**DOI:** 10.3389/fpls.2024.1470653

**Published:** 2025-01-08

**Authors:** Huan-Huan Lu, Yu-Ying Zheng, Yong-Sen Qiu, Liu-Ban Tang, Yan-Cui Zhao, Wen-Gang Xie

**Affiliations:** State Key Laboratory of Herbage Improvement and Grassland Agro-Ecosystems, Key Laboratory of Grassland Livestock Industry Innovation, Ministry of Agriculture and Rural Affairs, College of Pastoral Agriculture Science and Technology, Lanzhou University, Lanzhou, China

**Keywords:** *Elymus sibiricus* L., functional plant ecology, global warming, ensemble ecological niche models, potential spatiotemporal distribution pattern, priority planting region prediction, adaptability evaluation of introduction

## Abstract

The natural grassland in China is facing increasingly serious degradation. *Elymus sibiricus* L., as an important native alpine grass, is widely used in the restoration and improvement of natural grassland. In this study, the geographical distribution and environmental data of *E. sibiricus* in China were collected, and the potential spatiotemporal distribution pattern, planting pattern, and introduction adaptability of *E. sibiricus* were comprehensively predicted by using ensembled ecological niche model and Marxan model. The results show that (1) the potential spatial distribution of *E. sibiricus* mainly spans 33°–42°N and 95°–118°E. It was widely distributed in Qilian Mountains (northeast of Qinghai-Tibet Plateau), Taihang Mountains (junction of Loess Plateau and Inner Mongolia Plateau), and Tianshan Mountains; (2) with the passage of time, the suitable distribution regions of *E. sibiricus* generally showed a collapse trend, but its main distribution regions did not show obvious change, and the suitable regions (centroid) generally migrated to the southwest of China by 2.93 km; (3) the spatial distribution of *E. sibiricus* in the current period was significantly affected by the annual range of monthly near-surface relative humidity, mean annual air temperature, annual range of monthly potential evapotranspiration, mean monthly potential evapotranspiration, annual range of monthly climate moisture index, elevation, exchangeable Ca^2+^, available P, mean monthly near-surface relative humidity, exchangeable H^+^, and annual precipitation amount, respectively; (4) the potential planting area of *E. sibiricus* cover 2.059 × 10^5^ km^2^, which was mainly distributed in Qilian Mountains (northeast of Qinghai-Tibet Plateau), Taihang Mountains (southeast of Inner Mongolia Plateau), the middle part of Tianshan Mountains, and the southeast of Altai Mountains; (5) the introduction adaptability regions of six *E. sibiricus* germplasm (LM01–LM06) were all distributed in the high-elevation regions of western China. The study aims to provide an effective theoretical basis for the collection, preservation, and utilization of *E. sibiricus* germplasm resources in China.

## 1 Introduction

Grasslands, covering 40% of the earth’s surface and 69% of agricultural land, provide a wide range of ecosystem services for human beings, such as food production, water supply and regulation, carbon storage and climate mitigation, pollination, and a series of economic and cultural services (Bardgett et al., 2021). The area of all kinds of grassland in China is about 2.65 × 10^6^ km^2^, accounting for 27.6% of the total land (He et al., 2024). Due to human activities and natural factors, as many as 50% of grasslands worldwide have experienced varying degrees of degradation, nearly 5% of grasslands have been seriously degraded, and nearly 90% of grasslands in northern China have been degraded to some extent (Li et al., 2022). In addition, in the recent 130 years, the global average surface temperature has risen by 0.85°C, and the surface temperature will continue to rise in the future (Wu et al., 2020a). The change in global climate will not only change the precipitation and species distribution pattern but also further reduce the coupling degree between grassland structure and function (O’Mara, 2012). Grassland degradation has a profound impact on productivity decline, biodiversity loss, land degradation, and decline in ecosystem services.

Siberian wildrye (*Elymus sibiricus* L.), belonging to the genus *Elymus* of Poaceae, is a perennial, self-pollinated, allotetraploid (StStHH, 2*n* = 4*x* = 28) native alpine grass (Wang et al., 2017b; Xiong et al., 2021). As a model plant of *Elymus*, *E. sibiricus* is the dominant and constructive species in alpine meadow grassland in Qinghai-Tibet Plateau (QTP) (Liu et al., 2015), and its habitat is mainly composed of valley meadow, mountain meadow grassland, sparse forest, shrub, and open space in forest grassland (Yan et al., 2007). *E. sibiricus* has excellent cold resistance, drought resistance, strong adaptability, and high hay quality (Zhang et al., 2016; Wang et al., 2017a), which is widely used in the establishment of high-yield artificial grassland and the restoration of natural grassland in China. According to the Analysis Report on Supply and Demand of Grass Seeds in China in 2024, the total demand for various grass seeds in China in 2024 is 180,000–200,000 tons, including 70,000–80,000 tons for ecological restoration. In 2023, 16,500 tons of seeds of various ecological grasses were produced in China, among which the *Elymus* seed yield (including *E. sibiricus*) was 10,500 tons, accounting for 63.63% of the national ecological grass seed yield (https://www.forestry.gov.cn/c/www/lcdt/528077.jhtml). Ecological grass not only plays their ecological restoration functions but also is important forage grass in traditional pastoral areas, and there is a systematic and serious shortage of ecological grass seed (Wu et al., 2020b; Li et al., 2021). Therefore, *E. sibiricus* plays an important role in animal husbandry and ecological maintenance, and its utilization and management are in urgent need of scientific guidance.

Ecological niche models (ENMs), known as species distribution models (SDMs), use the known geographical distribution and environmental data of species to estimate the potential ecological niche of species and project it into the prediction regions, presenting the habitat characteristics of species in the form of probabilities (Miller, 2010), which is one of the important means of predicting the potential distribution of species. With the deepening of mathematical theory in ecological application research, the types of ENMs have gradually increased, such as generalized linear model (GLM), maximum entropy model (MAXENT), and artificial neural network model (ANN) (Schmitt et al., 2017). Stacked species distribution models integrate multiple ENMs to eliminate deviations and uncertainties among different algorithms and obtain more robust results and insights (Fan et al., 2024). Marxan model is an intelligent decision-making tool based on the concept of system conservation planning, which constructs a cost-effective optimization network according to the management objectives and costs of research objects to meet the biodiversity conservation objectives (Watts et al., 2017). ENMs and Marxan models are widely used in the prediction of potential geographical distribution of species, the assessment of biodiversity conservation, and the analysis of ecological characteristics (Merow and Silander, 2014; Zhang and Li, 2022).

At present, the research on *E. sibiricus* mainly focuses on biological characteristics (Xie et al., 2017; Li et al., 2020), genetic diversity (Ma et al., 2012; Zhang et al., 2015, 2018), variety breeding (Zhou et al., 2011), cultivation, and utilization (Yang et al., 2015; Liu et al., 2012), but the integration of potential spatiotemporal distribution pattern and intelligent selection of planting regions of *E. sibiricus* in China was largely unexplored. Based on ecological theory, this study predicted the potential distribution, planting pattern, and introduction adaptability of different *E. sibiricus* germplasm, in order to answer four scientific questions: (1) the potential spatiotemporal distribution pattern characteristics of *E. sibiricus* in China, (2) the potential planting pattern of *E. sibiricus* in China, (3) The main environmental factors affecting the distribution pattern of *E. sibiricus*, and (4) the characteristics of introduction adaptability regions of different *E. sibiricus* germplasm. This study aims to provide theoretical guidance for the utilization, protection, and management of *E. sibiricus* germplasm resources in China.

## 2 Materials and methods

### 2.1 Compilation and preprocessing of species distribution data

Based on the Chinese and Latin literary names of *E. sibiricus*, the distribution points in China were comprehensively compiled from the existing literature, field investigation, and public online database. Four online databases (GBIF, Ecoengine, iNaturalist, and iDigBio) related to plant distribution information were queried by the R package spocc v1.2.2 (https://cran.R-project.org/package=spocc), and the search ended on 1 June 2024. The species name and its classification were queried and corrected through the NCBI Taxonomy database using the R package taxize v0.9.1 (https://CRAN.R-project.org/package=taxizedb). The distribution data were checked for the common spatial and temporal errors using the R package CoordinateCleaner v3.0.1 (Zizka et al., 2019). To reduce spatial autocorrelation and sampling bias, the Spatially Rarefy Occurrence Data for SDMs tool in SDM Toolbox v2.4 (Brown et al., 2017) was used to rarefy the species distribution data to ensure that only one distribution point was contained in each environmental unit at 5 arc-min resolution (~10 km).

### 2.2 Collection and preprocessing of environmental variable data

To effectively explore the potential spatiotemporal distribution pattern of *E. sibiricus*, this study obtained environmental variables (**Table 1**) that widely affect the plant growth and distribution, including bioclimatic, climatic, soil, and topographic data. Bioclimatic and climatic data were sourced from Paleoclim website (http://www.paleoclim.org). Current bioclimatic and climatic data (CP, 1979–2013) were obtained using the Anthropocene v1.2b dataset, while future bioclimatic and climatic data (FP, 2071–2100) were obtained using the CMIP6 dataset under the MPI-ESM1-2-HR climate model (Jägermeyr et al., 2021) and the ssp375 economic sharing pathway (Riahi et al., 2017). These bioclimatic and climatic variables mainly represent long-term annual trends, seasonality, and extreme situations of temperature and precipitation. The biomass of grass roots was mainly distributed in 0–30 cm soil layer to absorb soil nutrients, and the fibrous root system in 0–10 cm soil layer was extremely important for growth and development (Li et al., 2011). Soil layer data (i.e., 0–0.045 m, 0.045–0.091 m) containing nutrients necessary for plant growth were obtained from the World Soil Database (http://globalchange.bnu.edu.cn/research/soilw) (Shangguan et al., 2014), and the average value of different soil layer data was calculated by ArcGIS v10.8 (https://desktop.arcgis.com/). Elevation indirectly affects the adaptability of species to the environment by affecting temperature, and the SRTM elevation data was selected from the WorldClim website (https://www.worldclim.org). All environmental variables were unified into GCS_WGS_1984, resampled to 5 arc-min resolution and masked to China region by ArcGIS software. The China region was created from the map with review number GS (2019) 1822, and the base map was not modified. In order to avoid the multicollinearity of environmental variables leading to overfitting of the prediction model, the Correlations and Summary Stats tool in SDM Toolbox was used to detect the correlation among environmental variables, retaining only environmental variables with |*r*| <0.80 and significant ecological significance. The reserved environment variables were normalized through Circuitscape tool v1.0.2 (http://www.circuitscape.org) as a reference with elevation data.

**Table 1:** Environmental variables for predicting the potential spatiotemporal distribution pattern of *E. sibiricus*.

Environment variable types	Environmental factors	Description
Bioclimatic variables	Bio01	Mean annual air temperature
	Bio02	Mean diurnal air temperature range
	Bio03	Isothermality
	Bio04	Temperature seasonality
	Bio05	Mean daily maximum air temperature of the warmest month
	Bio06	Mean daily minimum air temperature of the coldest month
	Bio07	Annual range of air temperature
	Bio08	Mean daily mean air temperatures of the wettest quarter
	Bio09	Mean daily mean air temperatures of the driest quarter
	Bio10	Mean daily mean air temperatures of the warmest quarter
	Bio11	Mean daily mean air temperatures of the coldest quarter
	Bio12	Annual precipitation amount
	Bio13	Precipitation amounts of the wettest month
	Bio14	Precipitation amounts of the driest month
	Bio15	Precipitation seasonality
	Bio16	Mean monthly precipitation amount of the wettest quarter
	Bio17	Mean monthly precipitation amount of the driest quarter
	Bio18	Mean monthly precipitation amount of the warmest quarter
	Bio19	Mean monthly precipitation amount of the coldest quarter
Climatic variables	GSP	Accumulated precipitation amounts on growing season days TREELIM
	NGD0	Number of growing degree days
	GDD0	Growing degree days heat sum above 0°C
	CMI_mean	Mean monthly climate moisture index
	CMI_range	Annual range of monthly climate moisture index
	HURS_mean	Mean monthly near-surface relative humidity
	HURS_range	Annual range of monthly near-surface relative humidity
	PET_mean	Mean monthly potential evapotranspiration
	PET_range	Annual range of monthly potential evapotranspiration
	NPP	Net primary productivity
	SCD	Snow cover days
Soil variables	PH	pH Value (H_2_O)
	SOM	Soil Organic Matter
	TN	Total N
	TP	Total P
	TK	Total K
	AN	Alkali-hydrolysable N
	AP	Available P
	AK	Available K
	CEC	Cation Exchange Capacity (CEC)
	EH	Exchangeable H+
	EAl	Exchangeable Al3+
	ECa	Exchangeable Ca2+
	EMg	Exchangeable Mg2+
	EK	Exchangeable K+
	ENa	Exchangeable Na+
Topographic variables	Elev	Elevation

### 2.3 Analysis of potential spatial distribution pattern of species

To ensure the consistency of environmental data and the effectiveness of model predictions, this study constructed three types of environmental datasets: the full environmental dataset under the current period (CP-full dataset), including bioclimatic, climatic, soil, and topographic data; the simplified environmental dataset under the current or future period (CP or FP-simp dataset), including bioclimatic, soil, and topographic data. The CP-full dataset is used to finely predict the potential spatial distribution pattern of *E. sibiricus* in the current period; the CP or FP-simp dataset is used to analyze the temporal pattern dynamic changes of the potential distribution of *E. sibiricus* from the current to future periods. The potential spatial distribution pattern of *E. sibiricus* was modeled and predicted by R package ssdm v0.2.9 (Schmitt et al., 2017), which integrated different environmental datasets and species distribution points. To obtain more robust prediction results, this study selected nine modeling algorithms, including MAXENT, GLM, generalized additive model (GAM), multiple adaptive regression splines (MARS), generalized boosting model (GBM), classification tree analysis (CTA), random forest (RF), and ANN and support vector machine (SVM).

During the modeling process, the pseudo-occurrence points of the species of *E. sibiricus* were automatically generated, and 75% distribution data were randomly divided into training datasets for model construction, and the remaining were used as testing datasets to evaluate model performance. The independence between training and testing datasets was verified by 10 repeated crossover methods based on holdout strategy. The area under the curve of receiver operating characteristics (AUC) threshold is 0.75 as the standard for ensemble modeling algorithms, and Pearson correlation tests were performed among algorithms. To coordinate the inherent advantages and disadvantages of each algorithm, an integrated scoring system of true skills statistics (TSS), Kappa, and AUC was used to evaluate the predictive performance of the ensemble model (Chau et al., 2023). The values of TSS, Kappa, and AUC ranged from 0 to 1, 0.85 ≤ TSS | Kappa ≤ 1.0 or 0.9 ≤ AUC ≤ 1.0 considered that the model was accurate; 0.70 ≤ TSS | Kappa < 0.85 or 0.8 ≤ AUC < 0.9, the model predicts well; 0.55 ≤ TSS | Kappa < 0.70 or 0.6 ≤ AUC < 0.7, the model predicts general; 0.40 ≤ TSS | Kappa < 0.55 or 0.5 ≤ AUC < 0.6, the model predicts poorly; if TSS | Kappa < 0.40 or AUC < 0.50, the model predicts abnormal. The relative importance of environmental variables was estimated by R package ssdm. Finally, the R package ssdm generates continuity distribution probability with a threshold accuracy of 100.

Based on the natural break classification method, the distribution probability was divided into three levels of suitability (high, low, and unsuitable regions) by using the Reclassify tool in ArcGIS software, and the area of different suitable levels in each administrative region was counted by using the Region analysis tool in ArcGIS software. To obtain the most suitable survival environment conditions for *E. sibiricus* in the current period, the species survival curve was calculated by MaxEnt v3.4.4 (Phillips et al., 2006). In the parameter settings of MaxEnt software, enable the options of Create response curves, Do jackknife, Random seed, and Write plot data; set Random test percentage to 25%, Regularization coefficient to 1.1, and Max number of background points to 10,000. The operation model was repeated 10 times based on the Bootstrap strategy and defaulted to other parameters.

### 2.4 Analysis of potential temporal distribution patterns of species

The change and migration of species to suitable regions were a response to environmental changes over a long period of time. To explore the temporal distribution pattern of *E. sibiricus* from the current (1979–2013) to future (2071–2100) period, Wilcoxon test was conducted on the area of suitable levels at different periods using SPSS v25.0 (https://www.ibm.com/spss). The geometric centroid of suitable regions is used as a general description of the distribution characteristics of species in each period, and the migration of the geometric centroid can more intuitively display the changes of suitable regions of species in different periods. To clarify the dynamic migration path of the suitable region of *E. sibiricus*, the distribution probability was used, the geometric centroid positions of *E. sibiricus* distribution in different periods were located by the region analysis tool in ArcGIS software and R package foreign v0.8 (https://cran.r-project.org/package=foreign), and the geographical distance between different centroids was calculated by R package geosphere v1.5 (https://cran.r-project.org/package=geosphere).

### 2.5 Analysis of potential planting patterns of species

The potential planting pattern of *E. sibiricus* in the current period was predicted by Marxan v4.0.5 (Game et al., 2013), which involves the setting of planning unit, target, cost, and parameter optimization. Considering the continuity of the planting region and the simulation ability of Marxan software, a square planning unit (grid) with a side length of about 5 km covering the land of China was created by using the Fishnet tool in ArcGIS software. According to the protection standard of the Guidelines for Management Planning of Protected Areas issued by the International Union for Conservation of Nature (IUCN, https://www.iucn.org/), 10% planting coverage rate was selected as the planting target, which does not affect the ecological environment and maintains the basic ecological functions of species. Due to Chinese policy of “arable land minimum” (Chen et al., 2019), this study only selects the probability of refined distribution in the current period and the consensus rate of herbaceous vegetation coverage as equally weighted planting cost. The consensus rate of herbaceous vegetation coverage was selected from Herbaceous Vegetation Reduced v1.0 dataset (https://www.earthenv.org/landcover). The parameters such as boundary length (BLM), species penalty factor (SPF), and iteration times were optimized by ArcGIS plug-in ArcMarxan Toolbox v2.0.2 (https://aproposinfosystems.com/). Based on the natural break classification method, the potential planting regions were divided into three levels of planting (high, low, and unselected regions) by using the Reclassify tool in ArcGIS software, and the area of different planting levels in each administrative region was counted by using the region analysis tool in ArcGIS software.

### 2.6 Prediction of introduction adaptability of different germplasms of *E. sibiricus*

In this study, two cultivars [*E. sibiricus* cv. “Tongde” (S1) and “Chuancao No. 2” (S2)] and six germplasms [LM01 (S3), LM02 (S4), LM03 (S5), LM04 (S6), LM05 (S7), and LM06 (S8)] of *E. sibiricus* were selected to carry out field experiments in the forage breeding base of the Grassland Science Research Institute, Hongyuan County, Sichuan Province (32°47’N, 102°32’E, 3460 m) for 3 years. The experiment adopted a randomized block design with three replicates, with a sample plot area of 15 m^2^ (3 m × 5 m) and a spacing of 0.5 m between plots. Each sample plot was evenly seeded with 33 g of seeds and the row spacing of 0.3 m. To effectively evaluate the growth performance of *E. sibiricus*, 23 agronomic traits (**Table 2**) for 8 tested materials were measured (*n* = 10) from 2021 to 2023 according to “Description Standard and Data Standard of *Elymus sibiricus* Germplasm Resources” (Li et al., 2023). The values of all agronomic traits were normalized by R package vegan v2.6 (https://CRAN.R-project.org/package=vegan) to unify dimensions. Principal components analysis (PCA) and phenotypic contribution analysis of all tested materials were carried out using R package FactoMineR v2.11 (https://CRAN.R-project.org/package=FactoMineR). The membership function analysis among tested materials was analyzed by SPSSPRO software (https://www.spsspro.com/) to evaluate the comprehensive properties of different materials. Based on the potential planting pattern of *E. sibiricus*, the top 25 municipal administrative regions were selected according to the decrease of potential planting area with high selected regions. The suitability probability was extracted by using the extraction analysis tool in ArcGIS software under constraints of different municipal administrative regions. The distribution probability was used as the geographical environment standard to measure the suitable distribution of *E. sibiricus*, and the agronomic traits were weighted by the phenotypic contribution degree to measure the adaptability level of different tested materials. The optimal dissimilarity index was screened by R package vegan, and a Mantel test between the top 25 municipal administrative regions and weighted agronomic trait values of six germplasms was carried out by ChiPlot online software (https://www.chiplot.online/) to predict the introduction adaptability of different germplasms.

**Table 2:** Measured agronomic traits indicators of *E. sibiricus*.

Character types	Agronomic traits	Description
Plant height traits	NPH	Natural plant height
	APN	Absolute plant height
Leaf traits	FLL	Flag leaf length
	FLW	Flag leaf width
	TSLL	Top second leaf length
	TSLW	Top second leaf width
Stem traits	SD	Stem diameter
	SNN	Stem node number
	RB	Reproductive branch
	NB	Nutrient branches
	TN	Tiller number
Panicle traits	EL	Ear length
	SN	Spikelet number
	FN	Flower number
Seed traits	SL	Seed length
	SW	Seed width
	TGW	Thousand grain weight
Production traits	FTLR	Fresh stem to leaf ratio
	DSLR	Dry stem to leaf ratio
	SY	Seed yield
	HY	Hay yield
	FGY	Fresh grass yield
	DFR	Dry to fresh ratio

## 3 Results

### 3.1 Data preprocessing and prediction performance evaluation of ENMs

**Figure 1:**
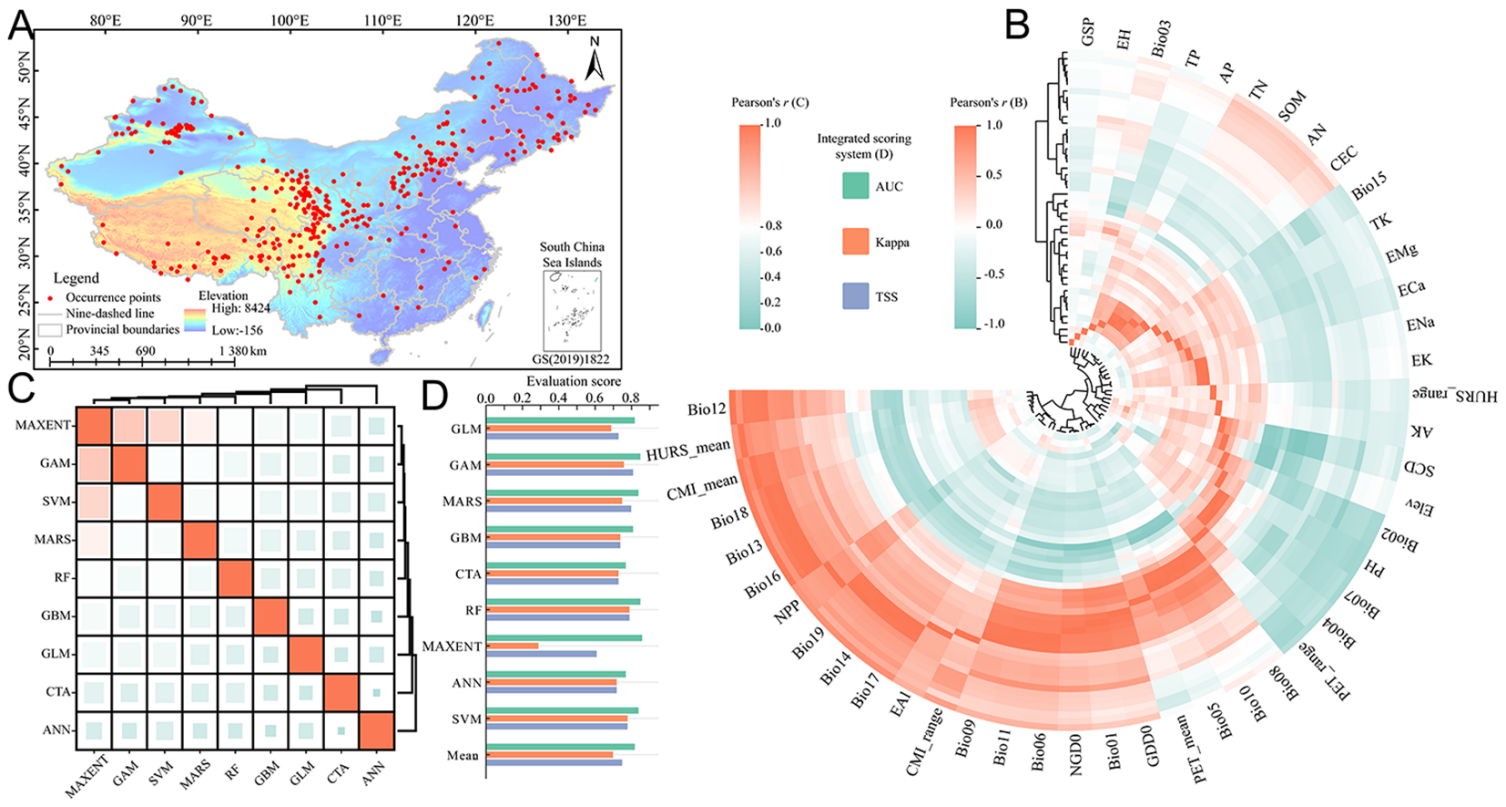
Refined modeling data processing and ecological niche model evaluation. **(A)** Geographical distribution records and study regions of *E. sibiricus*; **(B)** correlation among environmental variables in the current period; **(C)** the correlation among different algorithms in the current period; and **(D)** evaluation of integrated scoring system of different algorithms in current period. The meanings of abbreviations for environmental factors were shown in **Table 1**. MAXENT, maximum entropy; GLM, generalized linear model; GAM, generalized additive model; MARS, multiple adaptive regression splines; GBM, generalized boosting model; CTA, classification tree analysis; RF, random forest; ANN, artificial neural network; SVM, support vector machine.

A total of 538 distribution data of *E. sibiricus* were compiled in this study. After data quality control and rarefaction, there were 421 effective distribution points (**Figure 1A**), which were widely distributed in Qilian Mountains (northeast of QTP), Taihang Mountains (junction of Loess Plateau and Inner Mongolia Plateau), and Tianshan Mountains. The correlation of environmental variables showed that the correlation ranges of 46 environmental factors in the CP-full dataset were −0.98 to 0.99 (**Figure 1B**), and the correlation ranges of 25 environmental factors in the CP and FP-simp dataset were −0.98 to 0.99 and −0. 99 to 0.99 (**Supplementary Figure S1A**), respectively. Since 17 environmental factors in bioclimatic variables are derived from mean annual air temperature (bio01) and annual precipitation amount (bio12), bioclimatic variables are screened based on bio01 and bio12. After correlation analysis of all environmental factors, 24 environmental factors with low correlation and obvious ecological significance were screened out from the CP-full dataset, including Elev, AK, EAl, AP, ECa, EH, EMg, ENa, PH, TK, TN, TP, Bio01, Bio12, Bio15, Bio02, Bio03, Bio07, CMI_range, GSP, HURS_mean, HURS_range, PET_mean, and PET_range. The same eight environmental factors (Elev, Bio01, Bio12, Bio15, Bio02, Bio03, Bio07, and GSP) were screened from the CP or FP-simp dataset. The correlation analysis of nine modeling algorithms showed that the correlation range based on the CP-full dataset was 0.33 to 0.88 (**Figure 1C**); the correlation ranges based on the CP and FP-simp datasets were 0.54 to 0.93 (**Supplementary Figure S1B**) and 0.60 to 0.93 (**Supplementary Figure S1C**), respectively, indicating that there are significant deviations and uncertainties among different algorithms. The evaluation results of the integrated scoring system for the model prediction performance showed that RF algorithm had the best modeling effect on the spatial distribution of *E. sibiricus* under different environmental datasets, while MAXENT algorithm was the worst. It is worth noting that GLM algorithm cannot effectively model on CP or FP-simp dataset. Overall, the AUC, Kappa, and TSS based on the CP-full dataset were 0.82, 0.70, and 0.75, respectively (**Figure 1D**); the AUC, Kappa, and TSS based on the CP and FP-simp dataset were 0.89, 0.67, 0.72 and 0.89, 0.68, 0.72, respectively. The evaluation results of the integrated scoring system indicated that the ensemble model constructed by different algorithms can effectively simulate the potential spatial distribution pattern of *E. sibiricus*.

### 3.2 Importance evaluation and optimal survival value for environmental variables

**Figure 2:**
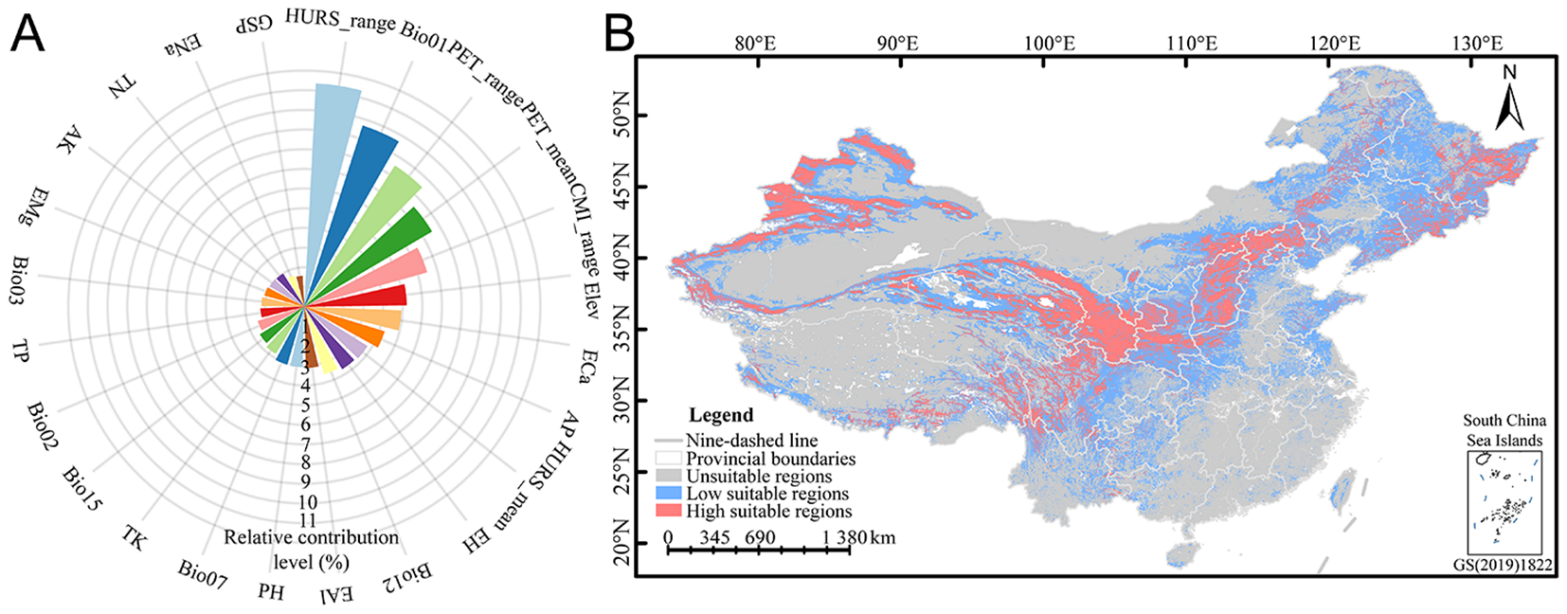
Relative contribution of environmental variables **(A)** and potential spatial distribution pattern **(B)** of *E. sibiricus*. The meanings of abbreviations for environmental factors were shown in **Table 1**.

Relative contribution can be used to evaluate the influence of environmental variables upon the spatial distribution of *E. sibiricus*. 24 environmental factors from the CP-full dataset were used to refine and predict the spatial distribution pattern of *E. sibiricus* in the current period. The relative contribution analysis showed that the relative contribution ranges of 24 environmental factors ranged from 1.57% to 11.36% (**Figure 2A**), among which the total relative contribution of bioclimatic, climatic, soil, and topographic variables was 23.62%, 39.23%, 31.93%, and 5.21%, respectively. Specifically, the cumulative relative contribution of 11 environmental factors reached 69.03%, including the annual range of monthly near-surface relative humidity (HURS_range, 11.36%), mean annual air temperature (Bio01, 9.67%), annual range of monthly potential evapotranspiration (PET_range, 8.52%), mean monthly potential evapotranspiration (PET_mean, 7.53%), annual range of monthly climate moisture index (CMI_range, 6.48%), elevation (Elev, 5.21%), exchangeable Ca^2+^ (ECa, 4.94%), available P (AP, 4.27%), mean monthly near-surface relative humidity (HURS_mean, 3.78%), exchangeable H^+^ (EH, 3.71%), and annual precipitation amount (Bio12, 3.57%). Overall, the spatial distribution pattern of *E. sibiricus* is obviously responsive to hydrothermal environmental conditions, especially humidity, air temperature, and potential evapotranspiration, and the elevation and soil calcium and phosphorus nutrients also play an important role in its distribution. In addition, based on the survival response curve, the optimum survival environment value under the optimum survival probability of *E. sibiricus* was extracted (**Table 3**). The ranges of optimum survival probability and its coefficient of variation under different environmental factors were 0.08–0.93 and 1.85%–125.47%, respectively, among which the optimum survival probability and coefficient of variation with GSP were extremely abnormal, indicating that GSP had no significant effect on the potential spatial distribution of *E. sibiricus*. According to the comprehensive analysis of optimum survival environment values for different types, it is concluded that the most suitable habitat of *E. sibiricus* is a semi-humid area with weak acidic soil rich in nitrogen, phosphorus, and potassium, small fluctuation of air temperature, low temperature, and high altitude.

**Table 3:** The optimal survival environment values under optimal survival probability of *E. sibiricus*.

Environmental factors	Mean survival probability (*n* = 10)	Coefficient of variation	Optimal survival value	Unit of optimal survival value
AK	0.46 ± 0.08	16.79%	357.31	mg/kg
AP	0.82 ± 0.03	3.07%	7.70	mg/kg
Bio01	0.82 ± 0.03	3.58%	2.00	°C
Bio02	0.69 ± 0.04	6.34%	13.65	°C
Bio03	0.74 ± 0.03	4.35%	0.36	°C
Bio07	0.75 ± 0.23	30.79%	59.08	°C
Bio12	0.75 ± 0.02	2.03%	487.66	kg·m^−2^·year^−1^
Bio15	0.79 ± 0.29	37.15%	26.68	kg·m^−2^
CMI_range	0.86 ± 0.03	2.92%	64.94	kg·m^−2^·month^−1^
EAl	0.76 ± 0.02	2.73%	0.10	me/100 g
ECa	0.81 ± 0.05	6.00%	14.53	me/100 g
EH	0.73 ± 0.03	4.52%	0.14	me/100 g
Elev	0.93 ± 0.02	2.48%	3355.44	M
EMg	0.87 ± 0.02	2.45%	2.79	me/100 g
Ena	0.73 ± 0.02	3.05%	0.56	me/100 g
GSP	0.08 ± 0.10	125.47%	343597.36	kg·m^−2^·gsl^−1^
HURS_mean	0.72 ± 0.01	1.85%	57.90	%
HURS_range	0.81 ± 0.03	3.62%	5.87	%
PET_mean	0.78 ± 0.03	3.28%	76.66	kg·m^−2^·month^−1^
PET_range	0.75 ± 0.01	1.98%	109.66	kg·m^−2^
PH	0.75 ± 0.02	2.67%	8.09	PH units
TK	0.73 ± 0.02	2.84%	2.11	g/100g
TN	0.69 ± 0.03	4.08%	0.44	g/100g
TP	0.71 ± 0.02	3.53%	0.09	g/100g

The meanings of abbreviations for environmental factors were shown in **Table 1**.

### 3.3 Potential spatial distribution pattern of *E. sibiricus*

Based on the natural break classification method, the potential spatial distribution of *E. sibiricus* was divided into three levels of suitability (**Supplementary Table S1**). The results showed that the potential spatial distribution mainly spans 33°–42°N and 95°–118°E (**Figure 2B**), which was concentrated in Qilian Mountains (northeastern of QTP), Taihang Mountains (the junction of Loess Plateau and Inner Mongolia Plateau), and Tianshan Mountains, while the rest was scattered in Altai Mountains, Kunlun Mountains (northern of QTP), Hengduan Mountains (central Yunnan-Guizhou Plateau), Qinling Mountains (south of Loess Plateau), and Changbai Mountains (southeast of Northeast China). This study predicted an area of 9.3784 million km^2^ in China, with high and low suitable regions accounting for 14.67% (1.3758 million km^2^) and 29.44% (2.7613 million km^2^), respectively. High suitable regions were mainly concentrated in western China, while low suitable regions were mainly distributed around high suitable regions. The high and low suitable regions involve 25 and 33 provincial administrative regions, respectively (**Supplementary Table S2**), among which the top five provincial administrative regions with the suitable area decreases in high suitable regions are Xinjiang (296,500 km^2^), Gansu (193,300 km^2^), Qinghai (125,700 km^2^), Sichuan (112,600 km^2^), and Heilongjiang (103,900 km^2^); the top 5 provincial administrative regions with the largest area of low suitable regions are Inner Mongolia (399,100 km^2^), Xinjiang (372,600 km^2^), Heilongjiang (270,300 km^2^), Qinghai (197,800 km^2^), and Sichuan (185,300 km^2^). Overall, the suitable area for *E. sibiricus* distribution accounts for about 44.11% of the total area in China, and it is concentrated in plateau regions.

### 3.4 Dynamic changes of the potential temporal distribution pattern of *E. sibiricus*

**Figure 3:**
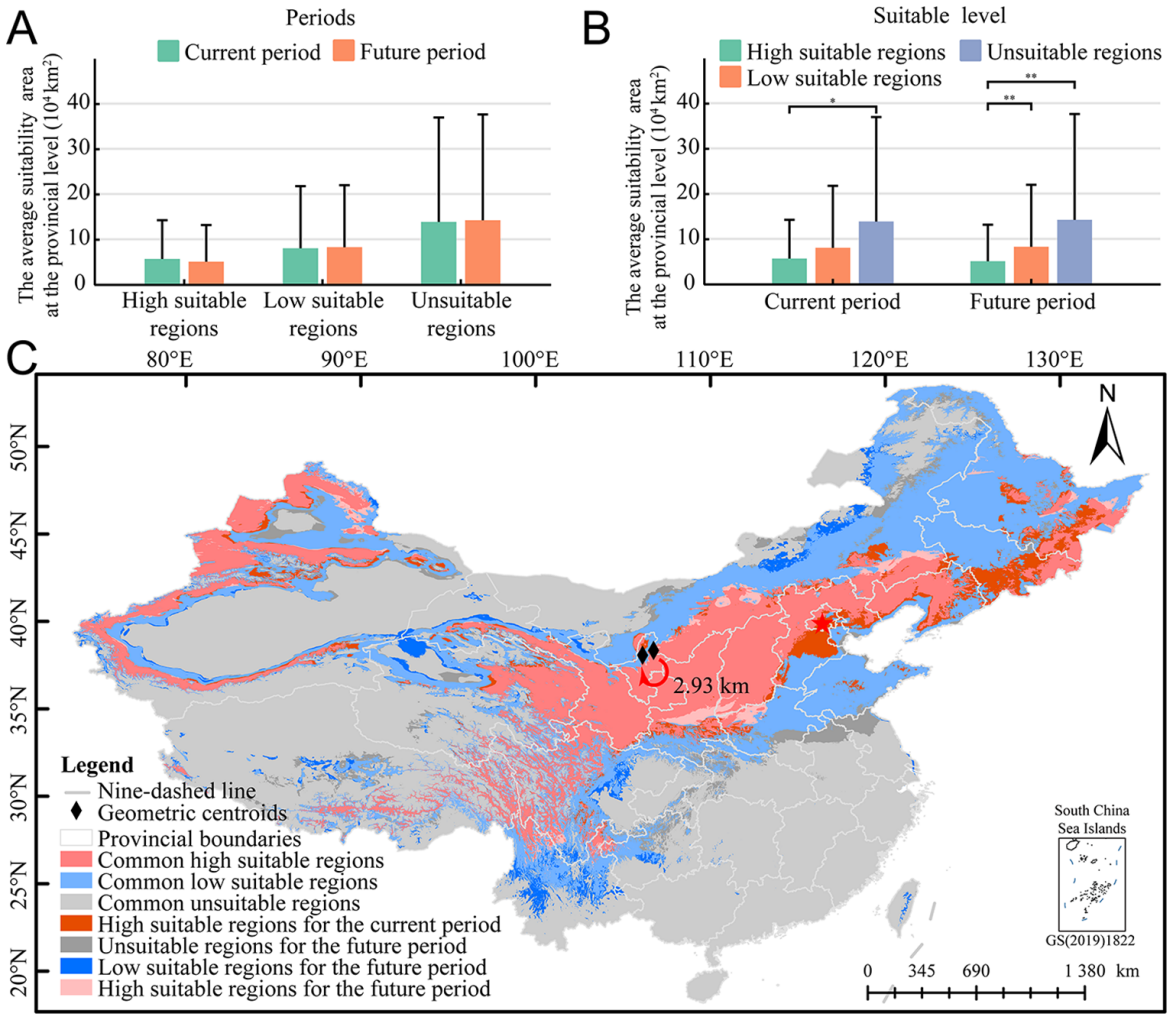
Dynamic changes in the potential temporal distribution pattern of *E. sibiricus*. **(A)** The area comparison of the same suitable level in different periods; **(B)** the area comparison of the same period in different suitable level; and **(C)** dynamic changes of potential distribution pattern of *E. sibiricus* in different periods. *p < 0.05, **p < 0.01.

The dynamic changes of the potential spatial distribution pattern of *E. sibiricus* across multiple periods were analyzed. The results showed that the levels of high, low, and unsuitable regions remained relatively stable across different periods (**Figure 3A**), but the area changes within each suitable level were significant during the same period (**Figure 3B**), indicating that the potential suitable pattern of *E. sibiricus* had migrated with the passage of time. Comparing the current period with the future period, the overall suitable area is estimated to grow between −2.72% and 4.65 million km^2^. Specifically, the area of high and low suitable regions is expected to increase by −10.66% (up to approximately 1.7684 million km^2^) and 2.89% (up to around 2.8817 million km^2^), respectively (**Figure 3C**). A deeper analysis of the dynamic changes within the different suitable levels revealed that over time, the percentage range of area growth for high and low suitable regions varied from −100.00% to 59.39% and from −86.56% to 1185.90%, respectively (**Figure 3C**, **Supplementary Table S3**). The regions predicted to experience relatively large expansions in their suitable areas are Guizhou, Yunnan, and Henan (high suitability), as well as Taiwan, Guangxi, Beijing, and Fujian (low suitability). Meanwhile, the areas expected to have relatively large contractions in their suitable regions are Shandong, Tianjin, and Chongqing (high suitability) and Zhejiang, Jiangsu, and Anhui (low suitability). Overall, compared to the present, the forecasted suitable area for *E. sibiricus* demonstrates a general decline; however, the key distribution centers remain relatively stable, such as Qilian Mountains (northeast of QTP), Taihang Mountains (junction of Loess Plateau and Inner Mongolia Plateau), and Tianshan Mountains. Furthermore, the centroid of the suitable habitat is estimated to shift southwestward by approximately 2.93 km (**Figure 3C**).

### 3.5 Potential planting distribution pattern of *E. sibiricus*

**Figure 4:**
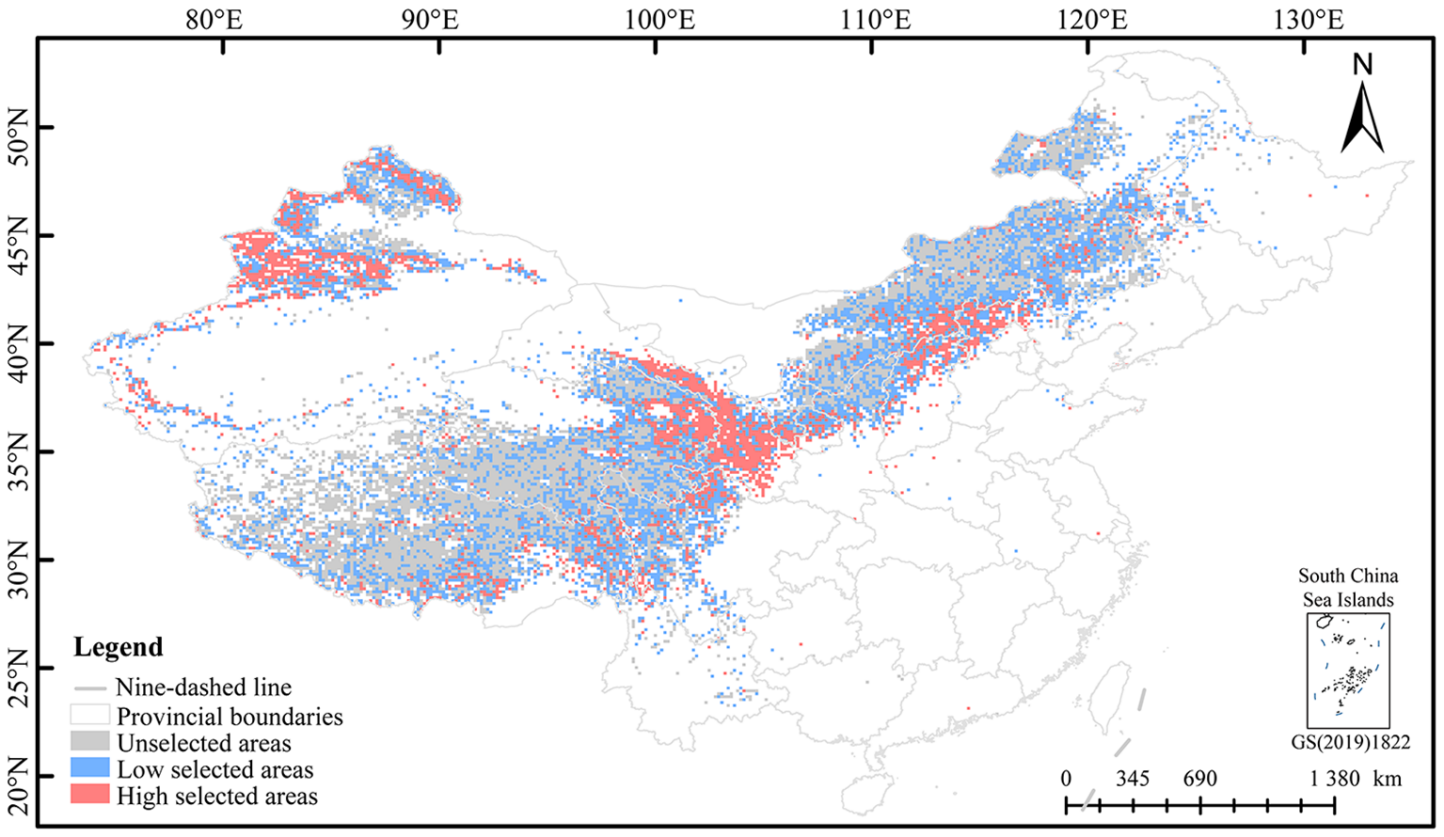
Distribution pattern of potential planting of *E. sibiricus*.

Based on the natural break classification method, the planting priority of *E. sibiricus* was divided into three levels of planting (**Supplementary Table S4**). This study only predicted the natural grassland area of 0.349 million km^2^ in China. The potential planting regions of *E. sibiricus* were mainly distributed in the Qilian Mountains (northeast of QTP), Taihang Mountains (southeast of Inner Mongolia Plateau), Tianshan Mountains, and Altai Mountains (**Figure 4**). The high and low selection planting areas were 66,600 km^2^ and 139,300 km^2^, respectively, among which the high and low selection planting regions involve 21 and 20 provincial administrative regions, respectively (**Supplementary Table S5**). The top 6 provincial administrative regions with the planting area decreases in high selection regions are Xinjiang (17,752.50 km^2^), Gansu (12,577.50 km^2^), Qinghai (7,582.50 km^2^), Tibet (7,335.00 km^2^), Inner Mongolia (7,155.00 km^2^), and Sichuan (4,275.00 km^2^). The top six provincial administrative regions with the largest area of low selection regions are Inner Mongolia (36,090.00 km^2^), Tibet (28,777.50 km^2^), Qinghai (20,970.00 km^2^), Xinjiang (20,205.00 km^2^), Sichuan (10,755.00 km^2^), and Gansu (6,007.50 km^2^). Overall, the suitable area for planting *E. sibiricus* accounts for 58.99% of the predicted natural grassland and 2.14% of the national area in China.

### 3.6 Introduction to the adaptability of different germplasm of *E. sibiricus*

**Figure 5:**
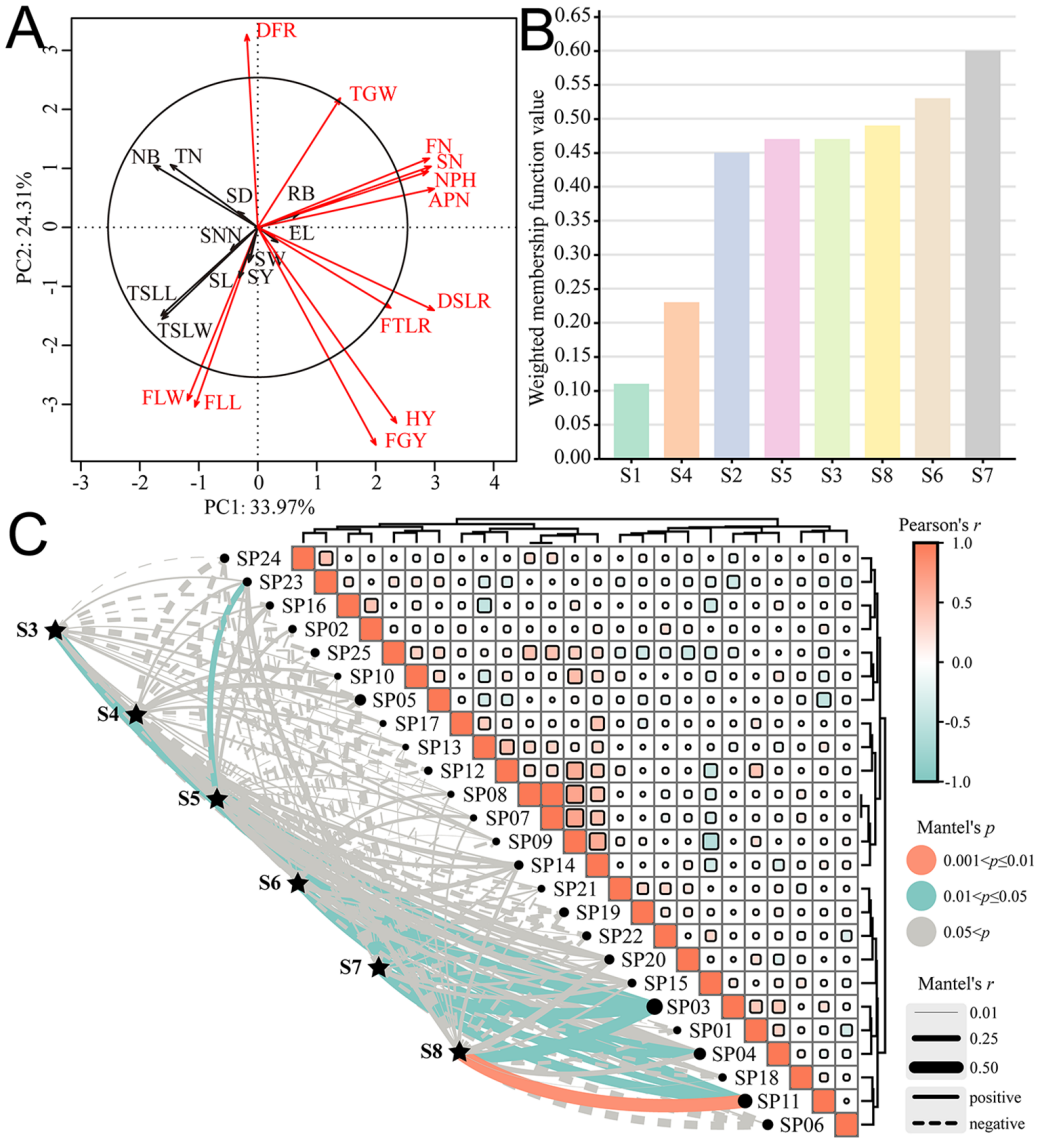
Comprehensive evaluation of different materials and introduction adaptability of *E. sibiricus*. **(A)** PCA and contribution analysis of agronomic traits; **(B)** Membership function evaluation of different tested materials; **(C)** Mantel Test analysis between tested materials and sample plots. The meanings of abbreviations for agronomic traits were shown in **Table 2**; SP01: Ili Kazakh Autonomous Prefecture; SP02: Tacheng region; SP03: Altay region; SP04: Changdu City; SP05: Zhangjiakou City; SP06: Aba Tibetan and Qiang Autonomous Prefecture; SP07: Zhangye City; SP08: Gannan Tibetan Autonomous Prefecture; SP09: Ulanqab City; SP10: Garze Tibetan Autonomous Prefecture; SP11: Hainan Tibetan Autonomous Prefecture; SP12: Dingxi City; SP13: Bayingolin Mongolian Autonomous Prefecture; SP14: Changji Hui Autonomous Prefecture; SP15: Xilingol League; SP16: Bortala Mongolian Autonomous Prefecture; SP17: Shigatse City; SP18: Haidong City; SP19: Shannan City; SP20: Lanzhou City; SP21: Chifeng City; SP22: Haixi Mongol and Tibetan Autonomous Prefecture; SP23: Wuwei City; SP24: Xinzhou City; SP25: Haibei Tibetan Autonomous Prefecture.

The PCA and contribution analysis of 23 agronomic traits showed that all agronomic traits explained 58.28% of the overall traits (**Figure 5A**), among which 12 agronomic traits, such as DFR, TGW, FN, SN, NPH, APN, DSLR, FTLR, HY, FGY, FLL, and FLW, contributed significantly to the comprehensive growth evaluation of *E. sibiricus*. The membership function analysis showed that, in comprehensive evaluation, the varieties S3 (0.47), S5 (0.47), S6 (0.53), S7 (0.60), and S8 (0.49) were superior to *E. sibiricus* varieties S1 (0.11) and S2 (0.45), while S4 (0.23) was superior to S1 but inferior to S2 (**Figure 5B**). The Mantel Test analysis of the introduction adaptability showed that the Pearson correlation range among all sample plots was −0.58 to 1.00 (**Figure 5C**); for example, the correlation among SP07, SP08, and SP09 was greater than 0.65, indicating that the plots had similar environmental conditions for adaptability distribution. Varieties S3, S5, S6, S7, and S8 had a significant positive correlation with SP03; S6, S7 with SP04; S3, S4, S6, S7, and S8 with SP11; and S5 with SP23 (*p* < 0.05). It was indicated that S3, S5, S6, S7, and S8 were suitable for planting in the southeast of the Altai Mountains represented by the Altay region (SP03); S6 and S7 were suitable for planting in the western section of the Hengduan Mountains represented by the Changdu City (SP04); S3, S4, S6, S7, and S8 were suitable for planting in the middle part of the Qilian Mountains represented by the Hainan Tibetan Autonomous Prefecture (SP11); and S5 was suitable for planting in the northern foot of the eastern part of the Qilian Mountains represented by the Wuwei City (SP23). Overall, the introduction adaptation regions of the six germplasms are all distributed in the high-altitude mountain region in western China, which indirectly verifies the rationality of the potential distribution and planting distribution pattern of *E. sibiricus*.

## 4 Discussion

### 4.1 Necessity of ensemble ENMs for prediction

In predicting the potential distribution probability of species, the single ENMs tends to overestimate in places with low species richness and underestimate in places with high species richness (Calabrese et al., 2014). The uncertainty of distribution prediction may distort policy making and planning. In the process of species distribution modeling, previous studies generally rely on multiple evaluation indicators to test the model results constructed by different algorithms based on the same data, selecting the algorithm with the highest statistical accuracy (Li and Wang, 2013; Xu et al., 2015). However, there may be inconsistencies among evaluation indicators. For example, in this study, the model with the highest AUC index was inconsistent with the model corresponding to the highest values of other indexes (Kappa and TSS). In addition, the construction of potential spatial distribution pattern of species is based on three prerequisites: (1) dynamic equilibrium of species distribution and environmental demand (Buckley et al., 2011), (2) idealized species migration ability (Xu et al., 2021), and (3) relatively conservative species ecological niche (Harris, 2015). The simulation results of the potential distribution of *E. sibiricus* in this study fails to effectively cover the existing distribution records, such as Wuyi Mountain, Luoxiao Mountain and Nanling Region, which show that the optimal algorithm may not achieve statistical optimum under all modeling conditions, nor may it effectively reduce the common sampling deviation (Randin et al., 2006). Nevertheless, to improve the accuracy of the model, using ensemble models to predict species distribution has become the trend of research (Grenouillet et al., 2011; Hao et al., 2020). The ensemble model maps the main trends of species distribution with the overall changes of all models and integrates the advantages of different algorithm models, such as the importance of variables or model response curve (Buisson et al., 2010), so as to effectively achieve and explore the prediction range of different algorithms and find consensus in the prediction of different mechanisms.

### 4.2 Key environmental factors affecting the potential spatial distribution pattern of *E. sibiricus*

In this study, the key environmental factors affecting the spatial distribution of *E. sibiricus* in the current period are all related to hydrothermal environmental conditions, especially humidity, air temperature, and potential evapotranspiration, which is consistent with the viewpoint that abiotic factors, mainly climate factors, dominated the species distribution pattern on a large scale (Soberon and Peterson, 2005). The response of plants to different environmental conditions gives them significant morphological plasticity (Sultan, 2000). Temperature and precipitation are the main climatic factors affecting forage growth (Li et al., 2018) and also the most important factors affecting genetic differentiation of the *Elymus* population (Yan et al., 2006). Studies have indicated that Bio01 and Bio12 have significant effects on the phenotype and genetic diversity of *Elymus* (Qi et al., 2013; Han et al., 2021); in this study, Bio01 and Bio12 were the main bioclimatic factors that significantly affect the potential spatial distribution of *E. sibiricus*. Compared to precipitation, temperature has a greater influence. Soil conditions can affect the growth and yield of aboveground and underground parts of plants (Liu et al., 2021). The appropriate dose of Ca^2+^ can activate the activity of antioxidant enzymes, alleviate the oxidative damage caused by low temperature to the cell membrane system, and thus improve the cold resistance of *E. nutans* (Qimei et al., 2021). Application of phosphate fertilizer can not only effectively increase the yield of artificial grassland build by *E. nutans*, but also prevent the loss of soil organic carbon (Renzneg et al., 2020). In this study, ECa and AP, as important soil factors, significantly affected the spatial distribution pattern. As an alpine plant, *E. sibiricus* obviously used ECa to improve its cold resistance and AP to increase seed yield to expand population size. Altitude is a comprehensive environmental factor, and the genetic diversity of the *Elymus* population increases first and then decreases with altitude, with the maximum value appearing at around 3,300 m (Yan et al., 2009). The most suitable altitude for *E. sibiricus* in this study is 3,355.44 m, which provides evidence for the hypothesis that alpine plants have higher genetic diversity at the most suitable altitude. In addition, environmental factors such as precipitation, temperature, and radiation intensity will change with different altitudes (Lv et al., 2023), while the relative contribution of elevation to the potential distribution of *E. sibiricus* in this study is only 5.21%, which indicates that elevation may not have a direct impact on the quality of life of species, but may indirectly affect the habitat suitability by affecting other environmental factors. These findings well prove that the key environmental variables screened in this study have practical significance.

### 4.3 Positive response of *E. sibiricus* population to climate change

The Qinghai-Tibet Plateau, located in southwest China, is the highest and largest plateau in the world, with an average elevation of over 4,000 m. During the uplift between Miocene periods and Quaternary periods, the geographical and climatic characteristics of QTP have undergone significant changes, making it recognized as one of the most important biodiversity hotspots in the world (Mao et al., 2021). QTP once served as a refuge for Northern temperate plants during the Quaternary period and was recognized as one of the important origin and radiation places of modern Northern temperate plants after the Glacial period (Xiong et al., 2022). The QTP is considered a potential center of origin for *E. sibiricus* population (Xiong et al., 2022, 2024). During the warming process from the last glacial period to Holocene, *E. sibiricus* gradually spread to Sichuan, Gansu, and Xinjiang and then further expanded to Qinghai and Inner Mongolia (Han et al., 2022; Xiong et al., 2022, 2024), which has formed a wide distribution pattern today. The pattern formed after the expansion of *E. sibiricus* population is similar to the potential distribution pattern predicted by this study, which is distributed in the vicinity of Qilian Mountains, Taihang Mountains, Kunlun Mountains, Hengduan Mountains, Qinling Mountains, and Changbai Mountains (Xiong et al., 2022, 2024). The potential planting and introduction adaptation areas of *E. sibiricus* predicted in this study yield similar results. In addition, geographical range is usually considered the basic determinant of species diversity and diversification (Ricklefs, 2006), and isolation (such as geographical, ecological, or behavioral isolation) is an important factor affecting the blocking mechanism of gene flow among populations, which has the possibility of leading to the formation of continent-island model, island model, and isolation-by-distance model (Qu et al., 2004). The potential distribution pattern predicted by this study may be caused by some historical geological events, thus forming the *E. sibiricus* population to gather and distribute in mountains and its vicinity. However, due to the lack of molecular data support, it is impossible to clarify specific historical geological events. Climate warming is a significant threat to global biodiversity, especially for endemic species in temperature-sensitive alpine areas (Verrall and Pickering, 2020). Temperature has an important influence on the productivity of alpine meadow plants, but the continuous rise of temperature may have a negative impact on alpine grasslands (Fazlioglu and Wan, 2021). Although some studies have suggested that many alpine plants in QTP-Hengduan Mountains may not face a high risk of extinction due to habitat contraction caused by climate warming, some species at extremely high altitudes show significant range contraction (Liang et al., 2018). In this study, with the climate warming and drought intensification in the future, the potential suitable regions of *E. sibiricus* show a collapse trend and migrate to the high-altitude areas in southwest China. The introduction adaptation regions of six *E. sibiricus* germplasm are also distributed in the high-altitude regions of mountains in west China. It is speculated that in response to global warming, the population of *E. sibiricus* will shrink and concentrate in the Qilian Mountains (northeast of QTP), Taihang Mountains (junction of the Loess Plateau and Inner Mongolia Plateau), and Tianshan Mountains. In addition, many studies have recorded the pattern of alpine species migrating to high altitude in response to climate warming (Dullinger et al., 2012; Körner and Hiltbrunner, 2021), which supports the “mountain-top” extinction and “uphill contraction” hypothesis (Sorte and Jetz, 2010), indicating that it is necessary to take protective measures for species inhabiting extremely high altitude or narrowly distributed.

## Data Availability

The original contributions presented in the study are included in the article/**Supplementary Material**. Further inquiries can be directed to the corresponding author.
