# Supplementary material for: Comprehensive prediction of potential spatiotemporal distribution patterns, priority planting regions, and introduction adaptability of Elymus sibiricus in the Chinese region
Source: Front Plant Sci. 2025 Jan 8;15:1470653. doi: 10.3389/fpls.2024.1470653 (PMC11751619; doi:10.3389/fpls.2024.1470653)
Supplement: Supplementary file 1 [file Table1.docx]

Supplementary Material

# 1 Supplementary Figures and Tables

## 1.1 Supplementary Figures


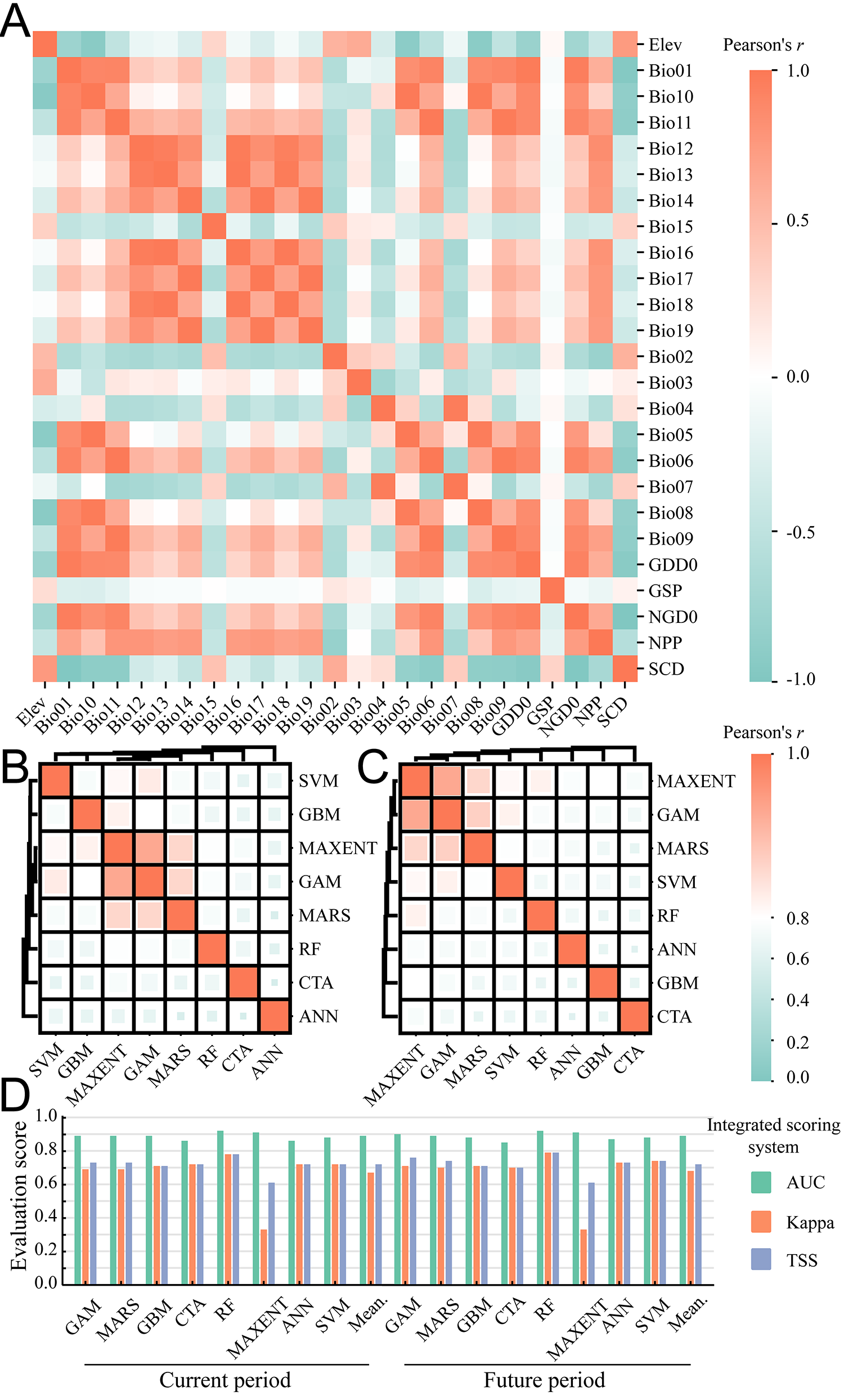


**Supplementary Figure 1.** **Prediction performance evaluation of ENMs.** (**A**) The environmental variables were correlation, and the upper and lower triangle were the environmental variables in the future and the current period respectively; (**B**) The correlation among different algorithms in the current period; (**C**) The correlation among different algorithms in the future period; (**D**) Evaluation of integrated scoring system of different algorithms in different periods.

## 1.2 Supplementary Tables

**Supplementary Table 1.** **The interruption value of different suitable levels based on the natural break classification method.** CP-full dataset: the full environmental dataset under the current period including bioclimatic, climatic, soil and topographic data; CP or FP-simp dataset: the simplified environmental dataset under the current or future period including bioclimatic, soil and topographic data.

| **Dataset types** | **The interruption value of different suitable levels** | | |
| --- | --- | --- | --- |
|  | **Unsuitable regions** | **Low suitable regions** | **High suitable regions** |
| CP-full dataset | 0.2035 | 0.4789 | 1.0000 |
| CP-simp dataset | 0.2209 | 0.4728 | 1.0000 |
| FP-simp dataset | 0.2330 | 0.4978 | 1.0000 |

**Supplementary Table 2.** **Potential distribution area of *E. sibiricus* under different suitable levels.**

| **Provincial administrative region** | **Area of participation (×10^4^ km^2^)** | **Potential distribution area under different suitable levels (×10^4^ km^2^)** | | |
| --- | --- | --- | --- | --- |
|  |  | **High suitable regions** | **Low suitable regions** | **Unsuitable regions** |
| Beijing City | 1.70 | 0.60 | 0.86 | 0.24 |
| Tianjin City | 1.11 | 0.01 | 0.38 | 0.72 |
| Hebei Province | 19.50 | 6.12 | 8.24 | 5.13 |
| Shanxi Province | 15.93 | 8.80 | 6.47 | 0.66 |
| Inner Mongolia Autonomous Region | 128.27 | 9.89 | 39.91 | 78.47 |
| Liaoning Province | 15.46 | 2.39 | 8.95 | 4.12 |
| Jilin Province | 21.05 | 4.55 | 13.17 | 3.33 |
| Heilongjiang Province | 54.06 | 10.39 | 27.03 | 16.64 |
| Shanghai City | 0.56 | 0.00 | 0.05 | 0.52 |
| Jiangsu Province | 8.79 | 0.03 | 3.61 | 5.15 |
| Zhejiang Province | 9.23 | 0.00 | 0.73 | 8.50 |
| Anhui Province | 12.86 | 0.01 | 3.11 | 9.74 |
| Fujian Province | 10.83 | 0.00 | 0.72 | 10.11 |
| Jiangxi Province | 14.79 | 0.00 | 0.45 | 14.34 |
| Shandong Province | 15.26 | 0.58 | 6.84 | 7.84 |
| Henan Province | 15.98 | 1.78 | 6.53 | 7.67 |
| Hubei province | 16.95 | 0.52 | 6.09 | 10.34 |
| Hunan Province | 19.04 | 0.01 | 1.51 | 17.52 |
| Guangdong Province | 15.29 | 0.00 | 0.94 | 14.35 |
| Guangxi Zhuang Autonomous Region | 20.31 | 0.13 | 3.02 | 17.16 |
| Hainan Province | 2.87 | 0.00 | 0.43 | 2.43 |
| Chongqing City | 7.62 | 0.29 | 3.01 | 4.32 |
| Sichuan Province | 45.51 | 11.26 | 18.53 | 15.72 |
| Guizhou Province | 15.71 | 0.67 | 6.72 | 8.32 |
| Yunnan Province | 34.12 | 2.03 | 10.26 | 21.83 |
| Xizang Autonomous Region | 109.07 | 7.78 | 17.58 | 83.71 |
| Shaanxi Province | 20.31 | 6.70 | 11.42 | 2.19 |
| Gansu Province | 43.50 | 19.33 | 9.36 | 14.81 |
| Qinghai Province | 65.65 | 12.57 | 19.78 | 33.30 |
| Ningxia Hui Autonomous Region | 5.23 | 1.49 | 2.54 | 1.20 |
| Xinjiang Uygur Autonomous Region | 168.10 | 29.65 | 37.26 | 101.20 |
| Taiwan Province | 3.12 | 0.00 | 0.65 | 2.47 |
| Hong Kong Special Administrative Region | 0.08 | 0.00 | 0.00 | 0.08 |
| Macau Special Administrative Region | 0.00 | 0.00 | 0.00 | 0.00 |

**Supplementary Table 3.** **Dynamic changes of potential spatial pattern of *E. sibiricus* in different periods.**

| **Province name** | **Area of participation (×10^4^ km^2^)** | **Current period** | | | **Future period** | | | **Rate of increase (Future period vs. Current period)** | | |
| --- | --- | --- | --- | --- | --- | --- | --- | --- | --- | --- |
|  |  | **Area of High  suitable regions (×10^4^ km^2^)** | **Area of Low  suitable regions (×10^4^ km^2^)** | **Area of  Unsuitable regions (×10^4^ km^2^)** | **Area of High  suitable regions (×10^4^ km^2^)** | **Area of Low  suitable regions (×10^4^ km^2^)** | **Area of  Unsuitable regions (×10^4^ km^2^)** | **High suitable regions** | **Low suitable regions** | **Unsuitable regions** |
| Beijing City | 1.73 | 1.61 | 0.13 | 0.00 | 0.86 | 0.87 | 0.00 | -46.51% | 591.05% | — |
| Tianjin City | 1.21 | 0.32 | 0.89 | 0.00 | 0.00 | 1.03 | 0.18 | -100.00% | 15.82% | — |
| Hebei Province | 19.67 | 14.78 | 4.89 | 0.00 | 9.59 | 9.83 | 0.25 | -35.15% | 101.13% | — |
| Shanxi Province | 15.99 | 15.24 | 0.75 | 0.00 | 15.67 | 0.32 | 0.00 | 2.83% | -57.44% | — |
| Inner Mongolia Autonomous Region | 129.08 | 19.96 | 61.85 | 47.27 | 21.10 | 58.52 | 49.46 | 5.72% | -5.38% | 4.62% |
| Liaoning Province | 15.64 | 10.83 | 4.82 | 0.00 | 7.57 | 7.94 | 0.13 | -30.03% | 64.90% | — |
| Jilin Province | 21.32 | 9.08 | 12.20 | 0.04 | 3.12 | 17.87 | 0.33 | -65.62% | 46.47% | 696.46% |
| Heilongjiang Province | 54.46 | 11.76 | 40.38 | 2.32 | 8.00 | 41.67 | 4.80 | -32.00% | 3.18% | 106.91% |
| Shanghai City | 0.64 | 0.00 | 0.00 | 0.64 | 0.00 | 0.03 | 0.61 | — | — | -4.15% |
| Jiangsu Province | 9.79 | 0.00 | 2.61 | 7.19 | 0.00 | 0.43 | 9.36 | — | -83.49% | 30.29% |
| Zhejiang Province | 9.52 | 0.00 | 0.03 | 9.50 | 0.00 | 0.01 | 9.51 | — | -71.47% | 0.20% |
| Anhui Province | 13.35 | 0.00 | 2.56 | 10.79 | 0.00 | 0.34 | 13.01 | — | -86.56% | 20.53% |
| Fujian Province | 10.98 | 0.00 | 0.00 | 10.98 | 0.00 | 0.00 | 10.98 | — | 400.00% | 0.00% |
| Jiangxi Province | 15.27 | 0.00 | 0.00 | 15.27 | 0.00 | 0.01 | 15.26 | — | — | -0.04% |
| Shandong Province | 15.59 | 0.57 | 15.02 | 0.00 | 0.00 | 14.48 | 1.10 | -99.40% | -3.60% | — |
| Henan Province | 16.15 | 1.57 | 11.69 | 2.88 | 2.26 | 9.11 | 4.77 | 43.78% | -22.07% | 65.71% |
| Hubei province | 17.56 | 0.08 | 4.64 | 12.84 | 0.00 | 1.97 | 15.59 | -97.48% | -57.64% | 21.43% |
| Hunan Province | 19.37 | 0.00 | 0.05 | 19.33 | 0.00 | 0.12 | 19.25 | — | 173.70% | -0.41% |
| Guangdong Province | 15.67 | 0.00 | 0.00 | 15.67 | 0.00 | 0.00 | 15.67 | — | — | -0.01% |
| Guangxi Zhuang Autonomous Region | 20.96 | 0.00 | 0.02 | 20.94 | 0.00 | 0.18 | 20.78 | — | 850.94% | -0.75% |
| Hainan Province | 2.91 | 0.00 | 0.00 | 2.91 | 0.00 | 0.00 | 2.91 | — | — | 0.00% |
| Chongqing City | 7.71 | 0.00 | 1.77 | 5.94 | 0.00 | 0.59 | 7.12 | -100.00% | -66.63% | 19.92% |
| Sichuan Province | 45.76 | 11.42 | 15.51 | 18.82 | 11.55 | 16.74 | 17.46 | 1.14% | 7.92% | -7.22% |
| Guizhou Province | 15.99 | 0.29 | 5.38 | 10.32 | 0.47 | 3.49 | 12.03 | 59.39% | -35.01% | 16.56% |
| Yunnan Province | 34.28 | 2.20 | 9.63 | 22.45 | 3.26 | 14.81 | 16.21 | 48.30% | 53.75% | -27.79% |
| Xizang Autonomous Region | 114.18 | 7.42 | 14.61 | 92.15 | 7.56 | 11.75 | 94.87 | 1.96% | -19.60% | 2.95% |
| Shaanxi Province | 20.35 | 13.80 | 6.33 | 0.22 | 13.78 | 5.45 | 1.13 | -0.16% | -13.99% | 415.21% |
| Gansu Province | 43.61 | 21.58 | 6.75 | 15.28 | 20.50 | 9.26 | 13.85 | -5.02% | 37.24% | -9.36% |
| Qinghai Province | 69.38 | 15.01 | 14.84 | 39.54 | 13.00 | 17.96 | 38.42 | -13.37% | 21.03% | -2.81% |
| Ningxia Hui Autonomous Region | 5.28 | 4.01 | 1.27 | 0.00 | 3.90 | 1.38 | 0.00 | -2.72% | 8.55% | — |
| Xinjiang Uygur Autonomous Region | 175.11 | 36.40 | 41.43 | 97.28 | 34.63 | 41.72 | 98.76 | -4.85% | 0.70% | 1.52% |
| Taiwan Province | 3.20 | 0.00 | 0.02 | 3.18 | 0.00 | 0.27 | 2.93 | — | 1185.90% | -7.91% |
| Hong Kong Special Administrative Region | 0.10 | 0.00 | 0.00 | 0.10 | 0.00 | 0.00 | 0.10 | — | — | 0.00% |
| Macau Special Administrative Region | 0.00 | 0.00 | 0.00 | 0.00 | 0.00 | 0.00 | 0.00 | — | — | 0.00% |

**Supplementary Table 4.** **The interruption value of different planting levels based on the natural break classification method.** Marxan: Potential planting probability based on Marxan model.

| Dataset types | The interruption value of different planting levels | | |
| --- | --- | --- | --- |
|  | Unselection regions | Low selection regions | High selection regions |
| Marxan | 28.0000 | 38.0000 | 68.0000 |

**Supplementary Table 5.** **Potential planting area of *E. sibiricus* under different planting levels.**

| **Provincial administrative region** | **Area of participation (km^2^)** | **Potential planting area under different planting levels (km^2^)** | | |
| --- | --- | --- | --- | --- |
|  |  | **High selected regions** | **Low selected regions** | **Unselected regions** |
| Beijing City | 67.50 | 45.00 | 22.50 | 0.00 |
| Tianjin City | 22.50 | 22.50 | 0.00 | 0.00 |
| Hebei Province | 6142.50 | 3127.50 | 2542.50 | 472.50 |
| Shanxi Province | 7155.00 | 3060.00 | 3397.50 | 697.50 |
| Inner Mongolia Autonomous Region | 81270.00 | 7155.00 | 36090.00 | 38025.00 |
| Co-owned by China and North Korea | 0.00 | 0.00 | 0.00 | 0.00 |
| Liaoning Province | 1012.50 | 90.00 | 585.00 | 337.50 |
| Jilin Province | 2272.50 | 472.50 | 720.00 | 1080.00 |
| Heilongjiang Province | 3510.00 | 360.00 | 1867.50 | 1282.50 |
| Shanghai City | 0.00 | 0.00 | 0.00 | 0.00 |
| Jiangsu Province | 22.50 | 22.50 | 0.00 | 0.00 |
| Zhejiang Province | 0.00 | 0.00 | 0.00 | 0.00 |
| Anhui Province | 22.50 | 0.00 | 22.50 | 0.00 |
| Fujian Province | 0.00 | 0.00 | 0.00 | 0.00 |
| Jiangxi Province | 0.00 | 0.00 | 0.00 | 0.00 |
| Shandong Province | 202.50 | 67.50 | 112.50 | 22.50 |
| Henan Province | 202.50 | 90.00 | 67.50 | 45.00 |
| Hubei province | 45.00 | 0.00 | 22.50 | 22.50 |
| Hunan Province | 0.00 | 0.00 | 0.00 | 0.00 |
| Guangdong Province | 45.00 | 22.50 | 0.00 | 22.50 |
| Guangxi Zhuang Autonomous Region | 67.50 | 0.00 | 0.00 | 67.50 |
| Hainan Province | 0.00 | 0.00 | 0.00 | 0.00 |
| Chongqing City | 0.00 | 0.00 | 0.00 | 0.00 |
| Sichuan Province | 21442.50 | 4275.00 | 10755.00 | 6412.50 |
| Guizhou Province | 202.50 | 45.00 | 90.00 | 67.50 |
| Yunnan Province | 3352.50 | 472.50 | 1260.00 | 1620.00 |
| Xizang Autonomous Region | 83542.50 | 7335.00 | 28777.50 | 47430.00 |
| Shaanxi Province | 7470.00 | 855.00 | 3735.00 | 2880.00 |
| Gansu Province | 20385.00 | 12577.50 | 6007.50 | 1800.00 |
| Qinghai Province | 56790.00 | 7582.50 | 20970.00 | 28237.50 |
| Ningxia Hui Autonomous Region | 4410.00 | 1170.00 | 2047.50 | 1192.50 |
| Xinjiang Uygur Autonomous Region | 49387.50 | 17752.50 | 20205.00 | 11430.00 |
| Taiwan Province | 0.00 | 0.00 | 0.00 | 0.00 |
| Hong Kong Special Administrative Region | 0.00 | 0.00 | 0.00 | 0.00 |
| Macau Special Administrative Region | 0.00 | 0.00 | 0.00 | 0.00 |
